# Advances in Studying Brain Morphology: The Benefits of Open-Access Data

**DOI:** 10.3389/fnhum.2017.00405

**Published:** 2017-08-04

**Authors:** Christopher R. Madan

**Affiliations:** Department of Psychology, Boston College Chestnut Hill, MA, United States

**Keywords:** structural MRI, neuroimaging, cortical structure, aging, cortical thickness, gyrification

Until recently, neuroimaging data for a research study needed to be collected within one's own lab. However, when studying inter-individual differences in brain structure, a large sample of participants is necessary. Given the financial costs involved in collecting neuroimaging data from hundreds or thousands of participants, large-scale studies of brain morphology could previously only be conducted by well-funded laboratories with access to MRI facilities and to large samples of participants. With the advent of broad open-access data-sharing initiatives, this has recently changed–here the primary goal of the study is to collect large datasets to be shared, rather than sharing of the data as an afterthought. This paradigm shift is evident as increase in the pace of discovery, leading to a rapid rate of advances in our characterization of brain structure. The utility of open-access brain morphology data is numerous, ranging from observing novel patterns of age-related differences in subcortical structures to the development of more robust cortical parcellation atlases, with these advances being translatable to improved methods for characterizing clinical disorders (see Figure 1 for an illustration). Moreover, structural MRIs are generally more robust than functional MRIs, relative to potential artifacts and in being not task-dependent, resulting in large potential yields. While the benefits of open-access data have been discussed more broadly within the field of cognitive neuroscience elsewhere (Van Horn and Gazzaniga, 2013; Poldrack and Gorgolewski, 2014; Van Horn and Toga, 2014; Vogelstein et al., 2016; Voytek, 2016; Gilmore et al., 2017), as well as in other fields (Choudhury et al., 2014; Ascoli et al., 2017; Davies et al., 2017), this opinion paper is focused specifically on the implications of open data to brain morphology research.

**Figure 1:**
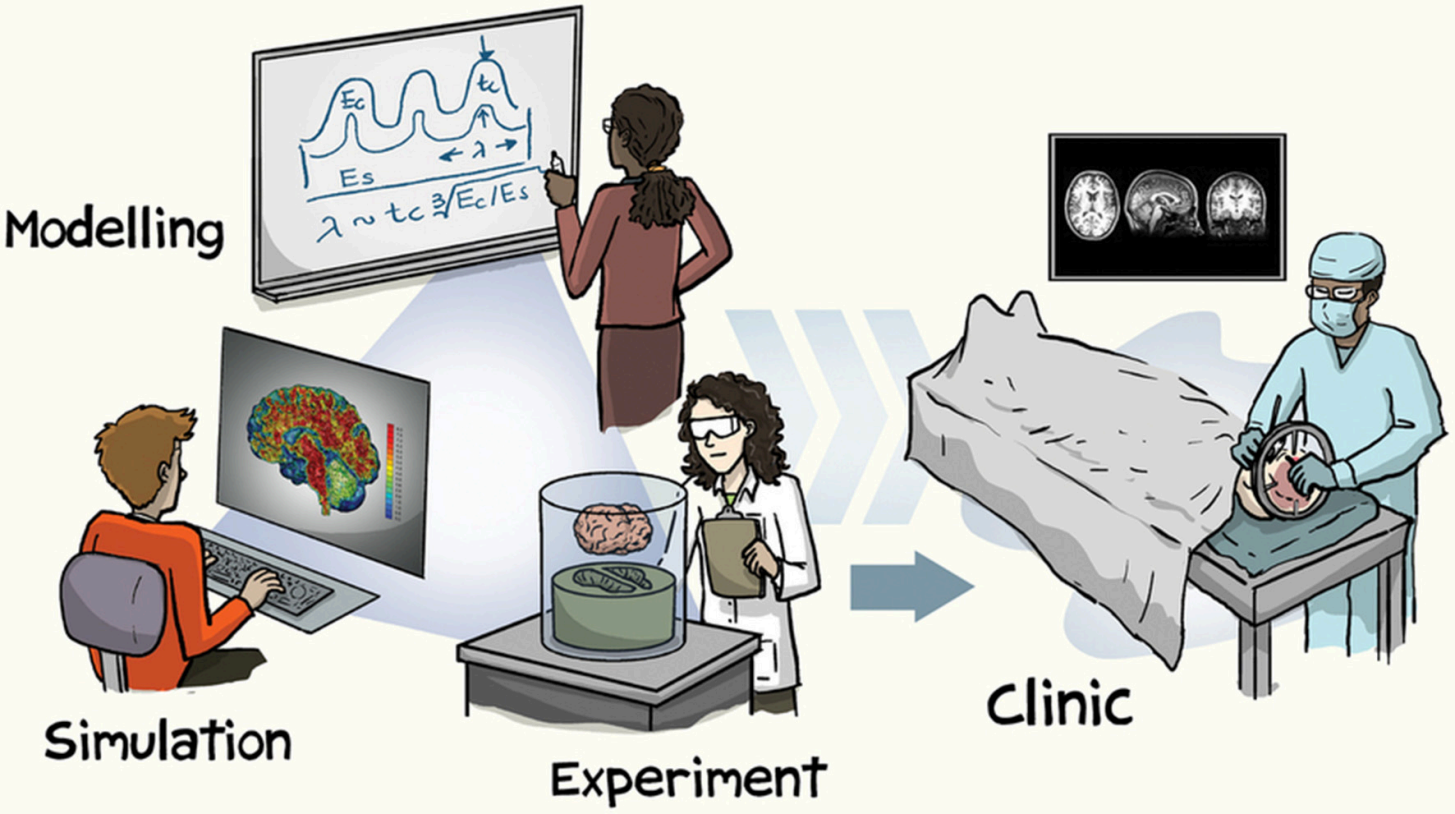
Illustration of approaches involved in brain morphology research. Reprinted with permission from Kuhl (2016), created by Jorge Cham. Copyright 2016, Nature Publishing Group.

## Why brain morphology?

Brain morphology is the study of the structural measures of the brain, e.g., volume and shape. Usually these measures are derived from T1 volumes, but other sequences such as T2 and FLAIR can also be useful. When comparing brains of individuals from patient populations with healthy controls, brain morphology can be used to identify differences in brain structure associated with the related medical condition (e.g., Alzheimer's disease or schizophrenia). Brain morphology can also be used to gain a better understanding normative brain development and aging (Frisoni et al., 2011; Falk et al., 2013; Fjell et al., 2014; Lee et al., 2014; Somerville, 2016; Lerch et al., 2017). Furthermore, brain morphology can beneficial in studying cognition, through an individual differences approach (Kanai and Rees, 2011).

As an example of studying memory using brain morphology, one could examine the relationship between behavioral measures of memory performance and structural measures such as hippocampal volume across a large number of individuals or as differences between participant groups (e.g., den Heijer et al., 2012; Ferreira et al., 2017; Olsen et al., 2017; Ritter et al., 2017). In contrast, researchers using fMRI to assess memory would examine differences in brain activity related to memory during encoding or retrieval tasks (i.e., subsequent memory effect [SME] or retrieval success [RS], respectively), looking for temporal fluctuations in regional activation in within-subject contrasts (e.g., Reagh and Yassa, 2014; Richter et al., 2016; Chen et al., 2017; de Chastelaine et al., 2017; Madan et al., 2017). Generally, both of these approaches can be useful, particularly when used as convergent approaches. For instance, while fMRI can provide within-subject estimates of regional brain activity, it is also influenced by age-related differences in BOLD signal variability (Grady and Garrett, 2013; Geerligs et al., 2017; Liu et al., 2017; Nomi et al., 2017), which can be at least partially attributed to effects of aging on neurovasculature (Thomas et al., 2014; Tsvetanov et al., 2015). In addition to aging, it has also been shown that genetic risk factors such as APOE can also be influence BOLD signal estimates (Filippini et al., 2009; Trachtenberg et al., 2012). Nonetheless, differences in brain morphology can, however, correspond to a myriad of inter-individual differences, including personality traits (Bjørnebekk et al., 2013; Holmes et al., 2016; Riccelli et al., 2017), genetic risk factors (Mormino et al., 2014; Strike et al., 2015; Chang et al., 2016), and age-related differences (Sowell et al., 2003; Allen et al., 2005; Fjell et al., 2009; Walhovd et al., 2011; Hogstrom et al., 2013; McKay et al., 2014; Madan and Kensinger, 2016; Cao et al., 2017). Generally, since brain morphology and fMRI studies are susceptible to different confounding factors, the use of both approaches as complementary methods is worth pursuing.

## Overview of available datasets

A number of datasets have been organized to advance the broad goal of improving our understanding of human brain structure. Two of the first well-used open-access datasets are Information eXtraction from Images (IXI) and Open Access Series of Imaging Studies (OASIS) (Marcus et al., 2007b, 2010). Briefly, the IXI dataset includes T1, T2, DTI, PD, and MRA data from nearly 581 healthy adults across the adult lifespan (20–86 years old). There are two OASIS datasets, one cross-sectional and one longitudinal. The OASIS cross-sectional dataset consists of T1 scans from 416 adults, aged 18–96, including over 100 adults that have been clinically diagnosed with Alzheimer's disease. The OASIS longitudinal dataset consists of T1 scans from 150 adults, aged 60–96, with at least two visits each and visits separated by at least 1 year; 64 adults were characterized as having dementia at their initial visit.

Currently, the most notable include Alzheimer's Disease Neuroimaging Initiative (ADNI) (Mueller et al., 2005; Jack et al., 2008, 2015; Weiner et al., 2015a), ADHD-200 Consortium (ADHD-200 Consortium, 2012; Bellec et al., 2017), Autism Brain Imaging Data Exchange (ABIDE) (Di Martino et al., 2014), SchizConnect (Ambite et al., 2015; Wang et al., 2016a), 1,000 Functional Connectomes Project (FCP) (Mennes et al., 2013), and the UK Biobank (Miller et al., 2016; Alfaro-Almagro et al., 2017). It is also important to acknowledge the data storage and computation infrastructure developed to manage this unprecedented amount of neuroimaging data, including software such as XNAT (Marcus et al., 2007a), COINS (Scott et al., 2011; Landis et al., 2016), INDI (Mennes et al., 2013; Kennedy et al., 2016), and LORIS/CBRAIN (Das et al., 2012; Sherif et al., 2014; Das et al., 2016), among others (Keator et al., 2009; Redolfi et al., 2009; Crawford et al., 2016).

For those particularly interested in relationships between brain structure and behavior in healthy individuals, the most relevant datasets are the the Human Connectome Project (HCP) (Van Essen et al., 2013; Glasser et al., 2016), Nathan Kline Institute - Rockland Sample (NKI-RS) (Nooner et al., 2012), Brain Genome Superstruct Project (GSP) (Holmes et al., 2015), and Cambridge Centre for Aging and Neuroscience (Cam-CAN) (Shafto et al., 2014; Taylor et al., 2017). Several large-scale developmental studies are also in-progress, including the Developing Human Connectome Project (dHCP) (Makropoulos et al., 2017) Adolescent Brain Cognitive Development (ABCD) study (https://abcdstudy.org), and Healthy Brain Network (Alexander et al., 2017). Additionally, a newly funded project, Lifebrain (http://www.lifebrain.uio.no), will be harmonizing data across eleven large-scale, brain imaging European cohorts, with data collection spanning seven countries and over 6,000 participants.

I currently maintain a list of open-access datasets of structural MRIs that includes further details of these datasets, along with additional datasets not described here, https://github.com/cMadan/openMorph.

## Working with open data

### Benefits

Apart from the obvious benefit of readily having access to datasets with sample sizes in the hundreds or more, several related benefits and cautions are also important to consider. An important consideration when collecting data for a study is financial cost (Guo et al., 2012; Mar et al., 2013; Poldrack and Gorgolewski, 2014). In this regard, the benefit of using open-access data is simple–the data has already been collected and is free to use. More related to the goals of a particular research question, open-access data can allow for **access to populations that may otherwise be unfeasible to recruit**–such as middle-age adults, patients, and individuals from other geographic regions. Many studies of aging often recruit young and older adults, but not middle-age adults. While a study's hypothesis may only bear on this comparison, it is also true that middle-age adults are more difficult to recruit (Lachman, 2015). Open-access datasets of aging often take a lifespan approach and do recruit middle-age adults, providing a continuous view of age-related differences in brain morphology. A population that is even harder to recruit from, at least for those without the relevant collaborators, is patient populations. Moreover, when patients are being recruited for a study, additional skills are necessary to appropriately characterize the patient's health and cognitive state–making the sharing of this data particularly valuable for further research, albeit with additional considerations related to the sharing of patient data (see Brakewood and Poldrack, 2013). Data sharing can also be viewed as minimizing the burden on participants, as a single MRI scan can be analyzed by multiple labs, rather than having multiple MRI scans of the same individual. More broadly, since many factors are known to influence brain morphology, it may be desirable to replicate analyses in other samples. Researchers are constrained in where they can recruit participants, but are also often located in areas where there is a so-called WEIRD (Western, Educated, Industrialized, Rich, and Democratic) demographic (Henrich et al., 2010). As such, it is important to also investigate the potential role of education (Kim et al., 2015; Steffener et al., 2016), socioeconomic status (Brito and Noble, 2014; Brito et al., 2017) and cultural backgrounds (Chee et al., 2011). However, this issue of recruitment can be circumvented by sharing data; for instance, many of the datasets included in the Consortium for Reliability and Reproducibility (CoRR) (Zuo et al., 2014) are from participants in China, which can enable researchers in western countries to reproduce their analyses using data from an East Asian sample.

Large open-access datasets, particularly those that are larger than would be commonly collected by a research lab, can further facilitate knowledge discovery by allowing for increased statistical sensitivity to **assess distributional properties within samples**. For instance, open-access data of patients with Alzheimer's disease has facilitated identifying heterogeneity within patient samples, allowing for the characterization of disease subtypes (Zhang et al., 2016; Dong et al., 2017), while other open-access data has helped establish consistent differences in brain morphology associated with schizophrenia (Moberget et al., 2017). These distribution-related insights are not limited to only characterizing patient populations, as recent findings have also demonstrated sex differences in the volume of many brain structures (Ritchie et al., 2017; Wierenga et al., 2017), with greater variability being found across males than females.

Beyond the discovery of new results directly, the sharing of open-access data is also beneficial to the development of **reproducible research methods**. In this regard, if everyone has access to the same data, researchers can more readily assess the influence of different analysis pipelines and approaches om morphological results. For instance, cortical thickness estimates produced by different software packages or the correspondence between manually traced structures relative to automated segmentation algorithms.

### Cautions and considerations

While the use of open-access data carries many benefits, they should not be used exclusively and to the detriment of future data collection. If specific datasets are solely used to characterize particular samples of individuals, this may result in **over-fitting to that particular sample** (e.g., if the findings of too many studies are based on a specific dataset). Relatedly, if care is not taken to assess the generalizability of findings, sample biases may become even more pronounced than before–e.g., instead of many researchers sampling participants from WEIRD demographics, they may be studying individuals from a specific location and set of inclusion criteria, despite the researchers themselves being located around the world.

It is also important to consider the **metadata collected along with the structural MRI data**. While age and sex demographic data will undoubtedly be included, some datasets stop here. If more data is collected, the secondary researcher needs to consider which datasets may be most suitable for the desired research question, as additional metadata–often cognitive or genetic data–will vary between dataests. Furthermore, many factors influence brain morphology estimates, such as head motion (Alexander-Bloch et al., 2016; Pardoe et al., 2016; Savalia et al., 2017) and circadian cycles (Nakamura et al., 2015), and additional consideration is needed to ensure that analyses are conducted appropriately, since the researchers using open data were not involved in data collection process.

When conducting analyses involving multiple datasets, or using data from a multi-site study, caution is also necessary in **‘harmonizing’ data across sites**. It is well-established that scanner effects can influence brain morphology estimates (Han et al., 2006; Jovicich et al., 2009, 2013; Iscan et al., 2015; Potvin et al., 2016; Madan and Kensinger, 2017b). Less obvious, however, are considerations related to the sample composition itself. For instance, studies may differ in their inclusion criteria–the presence of Axis-I disorder would result in exclusion for some datasets (e.g., HCP, GSP), but not others (e.g., NKI-RS). In other cases, the proportion of patients to controls may differ between studies, such as between ADNI and AIBL (Australian Imaging Biomarkers and Lifestyle Study of Aging) (Ellis et al., 2009).

## Recent advances

Beyond describing existing datasets and their related considerations, some examples of the utility of open-access datasets may be beneficial. The use of large open-access datasets have provided insights into differences in brain structure related to development (Mills et al., 2016) and aging (Cox et al., 2016; Madan and Kensinger, 2016, 2017a; Potvin et al., 2016, 2017; Wang et al., 2016b; DuPre and Spreng, 2017), as well as patient populations (relative to healthy controls) (Franke and Gaser, 2012; Gaser et al., 2013; Cole et al., 2015). These advances have been particularly evident for Alzheimer's disease, where the ADNI dataset has greatly contributed to our understanding of both healthy aging and dementia (Fjell et al., 2012; Zhang et al., 2012; Tamnes et al., 2013; Mormino et al., 2014; Wachinger et al., 2015; Weiner et al., 2015a,b; Wachinger et al., 2016; Coutu et al., 2017).

Providing more nuanced examples of the application of these datasets, they have also been used to develop an improved cortical parcellation atlas based on neuroanatomical landmarks (Klein and Tourville, 2012), as well as computational methods of estimating cortical parcellation and subcortical segmentation structure (Tustison et al., 2014; Redolfi et al., 2015; Wachinger et al., 2015, 2016; Madan and Kensinger, 2016, 2017a; Klein et al., 2017; Saygin et al., 2017). Datasets can also be used to measure the validity of standard morphological methods, such as the test-retest reliability of estimates of brain morphology (Madan and Kensinger, 2017b) and effects of head motion (Pardoe et al., 2016). Moreover, open-access data can be beneficial in methods development for tools designed for quality control and annotation (Heuer et al., 2016; Keshavan et al., 2017).

Despite a number of challenges involved in data sharing (Longo and Drazen, 2016; Mbuagbaw et al., 2017), open-access data is reshaping the field of neuroscience, as well as scientific research as a whole. The advent of open-access neuroimaging data suitable for brain morphology has recently and rapidly begun to move the field forward. In the coming years, I expect our understanding of the relationship between brain structure and inter-individual differences to increase drastically and meaningfully, supported by high-powered studies and the development of improved data analyses methods.

## Author contributions

The author confirms being the sole contributor of this work and approved it for publication.

### Conflict of interest statement

The author declares that the research was conducted in the absence of any commercial or financial relationships that could be construed as a potential conflict of interest.
